# Real-world outcomes of palbociclib plus endocrine therapy in elderly patients with HR+/HER2- advanced breast cancer in Japan: a subgroup analysis of the P-BRIDGE study by age group

**DOI:** 10.1007/s12282-025-01812-5

**Published:** 2026-02-11

**Authors:** Hiroko Masuda, Shigenori E. Nagai, Masaya Hattori, Tetsuhiro Yoshinami, Takuho Okamura, Kenichi Watanabe, Takahiro Nakayama, Michiko Tsuneizumi, Daisuke Takabatake, Michiko Harao, Hiroshi Yoshino, Natsuko Mori, Hiroyuki Yasojima, Chiya Oshiro, Madoka Iwase, Miki Yamaguchi, Takafumi Sangai, Shinsuke Sasada, Takanori Ishida, Manabu Futamura, Hirotaka Oda, Yasuaki Muramatsu, Norikazu Masuda

**Affiliations:** 1 Department of Breast Surgical Oncology, School of Medicine, Showa University, Tokyo, Japan; 2 Division of Breast Oncology, Saitama Cancer Center, Saitama, Japan; 3 Aichi Breast Clinic Hirabari, Nagoya, Japan; 4 Department of Breast Oncology, Aichi Cancer Center, Nagoya, Japan; 5 Department of Breast and Endocrine Surgery, Graduate School of Medicine, Osaka University, Osaka, Japan; 6 Department of Breast Oncology, Tokai University School of Medicine, Kanagawa, Japan; 7 Department of Breast Surgery, National Hospital Organization Hokkaido Cancer Center, Sapporo, Japan; 8 Department of Breast and Endocrine Surgery, Osaka International Cancer Institute, Osaka, Japan; 9 Department of Breast Surgery, Shizuoka General Hospital, Shizuoka, Japan; 10 Department of Breast Oncology, National Hospital Organization Shikoku Cancer Center, Matsuyama, Japan; 11 Department of Breast Oncology, Jichi Medical University, Shimotsuke, Japan; 12 Breast and Endocrinological Surgery, Ishikawa Prefectural Central Hospital, Kanazawa, Japan; 13 Department of Breast Surgery, Seirei Hamamatsu General Hospital, Hamamatsu, Japan; 14 Department of Surgery, Breast Oncology, National Hospital Organization Osaka National Hospital, Osaka, Japan; 15 Department of Breast Surgery, Kaizuka City Hospital, Osaka, Japan; 16 Department of Breast and Endocrine Surgery, Nagoya University Hospital, Nagoya, Japan; 17 Department of Breast Surgery, JCHO Kurume General Hospital, Kurume, Japan; 18 Department of Breast and Thyroid Surgery, Kitasato University School of Medicine, Sagamihara, Japan; 19 Department of Surgical Oncology, Research Institute for Radiation Biology and Medicine, Hiroshima University, Hiroshima, Japan; 20 Division of Breast and Endocrine Surgical Oncology, Tohoku University Graduate School of Medicine, Sendai, Japan; 21 Department of Breast Surgery, Gifu University Hospital, Gifu, Japan; 22 Oncology Medical Affairs, Pfizer Japan Inc., Tokyo, Japan; 23 Department of Breast Surgery, Graduate School of Medicine, Kyoto University, 54 Shogoin-kawahara-cho, Sakyo-ku, Kyoto, 606-8507 Japan

**Keywords:** Advanced breast cancer, Palbociclib, Elderly, Japanese patients, Real-world evidence

## Abstract

**Background:**

Palbociclib (PAL), a cyclin-dependent kinase 4/6 inhibitor, plus endocrine therapy (ET) is recommended as first-line (1L) treatment for hormone receptor-positive (HR+)/ human epidermal growth factor 2 negative (HER2-) advanced breast cancer (ABC). Despite broad use in real-world clinical settings, few studies have evaluated its effectiveness in elderly patients in Japan.

**Methods:**

The multicenter, observational P-BRIDGE study included HR+/HER2- ABC patients (*N* = 693) who initiated PAL + ET as 1L or second-line treatment during 2017–2020 in Japan. Treatment outcomes and patterns were evaluated by age category (< 65; ≥ 65 to < 75; ≥ 75 years).

**Results:**

Among patients treated with 1L PAL + ET (*N* = 426), 266, 118, and 42 patients were aged < 65, ≥ 65 to < 75, and ≥ 75 years, respectively. Patients aged ≥ 75 years were less likely to initiate PAL at 125 mg (64.3%) than patients aged < 65 (95.5%) and ≥ 65 to < 75 (88.1%) years. More patients aged ≥ 75 years discontinued PAL due to adverse events than other age groups. Median real-world progression-free survival (95% CI) was 24.5 months (18.2–30,4), 25.7 months (16.8–36.7), and 45.4 months (20.4–52.4), in the < 65, ≥ 65 to < 75, and ≥ 75-year age groups, respectively. Corresponding median overall survival was 68.2 months (65.0-not reached [NR]), NR (56.3-NR), and 68.0 months (45.8-NR), respectively.

**Conclusions:**

The effectiveness of PAL + ET in elderly patients in the real-world setting in Japan appears comparable to other age groups. These findings support PAL + ET as a viable treatment option for all patients, including the elderly, while highlighting the need for close monitoring and individualized treatment strategies, especially in older patients.

**Supplementary Information:**

The online version contains supplementary material available at 10.1007/s12282-025-01812-5.

## Introduction

Palbociclib, a first-in-class cyclin-dependent kinase 4 and 6 (CDK4/6) inhibitor, plus endocrine therapy (ET), is recommended as the standard of care for both first-line (1L) and second-line (2L) treatment of hormone receptor positive (HR+)/human epidermal growth factor receptor 2 negative (HER2-) advanced breast cancer (ABC) in local and international guidelines [1–3]. In the phase 3, randomized, double-blind, placebo-controlled PALOMA-2 and -3 studies, treatment with palbociclib + ET significantly improved progression-free survival (PFS) in patients with HR+/HER2- ABC (27.6 vs. 14.5 months; hazard ratio [HR] [95% CI]: 0.563 [0.461–0.687]; *p* < 0.0001; and 9.5 vs. 4.6 months; HR [95% CI]: 0.46 [0.36–0.59]; *p* < 0.0001, respectively) [4, 5]. Overall survival (OS) was also numerically longer with palbociclib + ET compared with ET monotherapy in PALOMA-2 and -3 [6, 7]. Median OS (95% CI) was 53.9 months (49.8–60.8) with palbociclib + letrozole versus 51.2 months (43.7–58.9) with letrozole monotherapy in PALOMA-2 [6], and 34.9 months (28.8–40.0) with palbociclib + fulvestrant versus 28.0 months (23.6–34.6) with fulvestrant monotherapy in PALOMA-3 [7]. These differences were not statistically significant (HR [95% CI]: 0.96 [0.78–1.18]; *p* = 0.34 and HR: 0.81 [95% CI: 0.64–1.03]; *p* = 0.09 in PALOMA-2 and PALOMA-3, respectively).

It is important to note that patients enrolled in randomized controlled trials (RCTs) must meet strict eligibility criteria for enrolment and are closely monitored under specialized conditions. Such restrictions can limit the generalizability of findings from RCTs to the real-world clinical setting, in which the drug is administered to a heterogeneous patient population with diverse backgrounds and characteristics. Furthermore, elderly patients are frequently under-represented in RCTs [8] despite facing unique challenges, such as an increased prevalence of comorbidities and the presence of multiple concomitant medications, which may complicate treatment selection [8–10]. In the pooled analysis of the PALOMA clinical studies, 48/444 (PALOMA-2) and 27/347 (PALOMA-3) patients in the palbociclib arm were aged ≥ 75 years [11]. Real-world evidence is, therefore, necessary to inform the treatment of patient populations that are either excluded from, or under-represented, in clinical trials.

To date, limited data exists regarding the relative efficacy of palbociclib across various age groups in Japan; however, there was no clinically relevant difference in PFS in a pooled analysis of the PALOMA clinical studies between age groups [11]. Large-scale, real-world data evaluating palbociclib in older patients has been reported internationally, including in the P-REALITY X study in the US and the PalomAGE study in France. These studies found no significant difference in effectiveness and safety with palbociclib in older women with ABC [12, 13]. However, given differences in the medical environment between Western countries and Japan, country-specific data on the effectiveness and safety of palbociclib treatment in elderly patients with ABC in clinical practice in Japan is needed. Additionally, due to the increasing aging population in Japan, additional data on palbociclib’s effectiveness and treatment patterns in elderly Japanese patients is warranted. To address this gap, we performed a subgroup analysis to evaluate treatment patterns and outcomes in patients with HR+/HER2- ABC across various age groups (< 65 years; ≥ 65 to < 75 years; ≥ 75 years) from the retrospective, multicenter, observational P-BRIDGE (Palbociclib in Japan: Breast cancer Real-world Investigation of DruG utilization and Effectiveness) study evaluating the real-world effectiveness of palbociclib plus ET in clinical practice in Japan.

## Patients and methods

### Study design and data source

A detailed description of the methodology for the P-BRIDGE study has been published previously [14]. Briefly, P-BRIDGE (NCT05399329) was a retrospective, multicenter, observational study conducted across eligible study sites in Japan to evaluate real-world clinical outcomes and treatment patterns of palbociclib in Japanese adults with HR+/HER2- ABC.

The study comprised a medical record review of 20 study sites to identify patients who had received palbociclib plus ET as 1–2L treatment between 15 December 2017 and 31 December 2020 (data cutoff date: 16 February 2024). The study was approved by the Institutional Review Board (IRB) at each study site and was conducted in accordance with Declaration of Helsinki, and all local laws. Informed consent was obtained from all individual participants included in the study.

### Patients

A detailed description of study participants in P-BRIDGE has been published previously [14]. Briefly, patients were aged ≥ 20 years with a diagnosis of HR+/HER2- ABC and had received palbociclib plus ET in the 1–2L setting. Patients were categorized by treatment line (1L; 2L) and age category (< 65 years; ≥ 65 to < 75 years; and ≥ 75 years). Treatment line was defined according to the Japanese Breast Cancer Society Clinical Practice Guidelines for systemic treatment of breast cancer, 2018 edition [2]. Patients were excluded if they had previously received 1L chemotherapy (CT). One induction CT regimen was permitted if its purpose was to reduce tumor burden, provided the patients had switched to ET before PD.

### Outcomes

The primary endpoint of the study was real-world PFS (rwPFS) of palbociclib plus ET, defined as the time from the start of palbociclib plus ET to physician-documented PD or death due to any cause, whichever occurred first. If there was no record of death or PD, patients with a history of treatment beyond palbociclib in their medical records were censored at the start date of the next treatment, and patients with no history of treatment beyond palbociclib in their medical records were censored at the date of their last visit during the study period (date of last confirmed survival).

Secondary endpoints included real-world chemotherapy-free survival (rwCFS), OS, baseline demographics, characteristics, and treatment patterns (initial dose, duration of treatment, and dose reductions and treatment discontinuations and their reasons). Real-world CFS was defined as the time from start of palbociclib treatment to the start of first subsequent chemotherapy or death due to any cause, whichever occurred first. If there was no clinical record of initiation of first chemotherapy (either oral or intravenous) or death, the date of the last visit was used as censoring. An additional rwCFS analysis was also performed, with rwCFS defined as the time from the start of palbociclib to the start of first subsequent intravenous (IV) CT or death due to any cause, whichever occurred first (IV-CFS). If there was no clinical record of initiation of first IV-CT or death, the date of the last visit was used as censoring. OS was defined as the time from the start of palbociclib treatment to death due to any cause. Additional exploratory OS analyses were also performed: (A) breast cancer-specific survival (BCSS) was defined as the time from the start of palbociclib treatment to death due to the breast cancer, with cases in which the death was due to diseases other than breast cancer or the cause of death was unknown censored. (B) OS following IV-CT was defined as the time from the start of IV-CT to death from any cause. If no clinical record of death was available, patients were censored at the date of their last visit during the study period.

Anticancer drug treatments initiated after palbociclib were also recorded as subsequent therapy.

### Statistical analysis

Patient outcomes were evaluated overall and by treatment line (1L; 2L) and age category (< 65 years; ≥ 65 to < 75 years; ≥ 75 years). Continuous variables were summarized descriptively, including means, standard deviations, medians, and ranges. Categorical variables were summarized using frequencies and proportions. Time to event analyses, including rwPFS, OS, and rwCFS, were assessed using the Kaplan-Meier method, with medians and 95% CIs presented.

All statistical analyses were performed using SAS^®^ version 9.4 (SAS Institute, Cary, NC, USA).

## Results

A total of 693 patients were enrolled in the P-BRIDGE study and were included in the present analysis, including 426 patients who received palbociclib plus ET as 1L and 267 patients who received palbociclib plus ET as 2L treatment (Fig. S1).

Among 1L patients, 266 (62.4%) were aged < 65 years, 118 (27.7%) were aged ≥ 65 to < 75 years, and 42 (9.9%) were aged ≥ 75 years. Among 2L patients, 161 (60.3%) were aged < 65 years, 66 (24.7%) were aged ≥ 65 to < 75 years, and 40 (15.0%) were aged ≥ 75 years. Of those aged ≥ 75 years, 11 in the 1L setting and 15 in the 2L setting were aged ≥ 80 years.

Demographic and clinical characteristics of patients receiving palbociclib plus ET as 1L and 2L treatment according to age group are presented in Table 1. In both the 1L and 2L treatment groups, the proportion of patients with comorbidities at the time of palbociclib initiation tended to increase with age. The proportion of patients with cardiac disease was highest in the ≥ 75-year age group in both the 1L and 2L treatment groups. In the 1L treatment group, the proportion of patients with bone metastases only, those with a treatment-free interval (TFI) < 12 months, and pre- and post-operative ET and CT decreased with increasing age. Conversely, the proportion of patients without symptoms at the time of palbociclib initiation increased with age. In the 2L treatment group, the proportion of patients with visceral metastases increased with age, but no other consistent trends were observed.

**Table 1:** Patient demographics and clinical characteristics

	First-line (N = 426)			Second-line (N = 267)		
	< 65 years	≥ 65 to < 75 years	≥ 75 years	< 65 years	≥ 65 to < 75 years	≥ 75 years
	n = 266	n = 118	n = 42	n = 161	n = 66	n = 40
Sex						
Male	1 (0.4)	1 (0.8)	1 (2.4)	1 (0.6)	0	0
Female	265 (99.6)	117 (99.2)	41 (97.6)	160 (99.4)	66 (100.0)	40 (100.0)
Menopausal status^a^						
Pre/perimenopausal	85 (32.1)	0	0	69 (43.1)	0	0
Postmenopausal	153 (57.7)	110 (94.0)	39 (95.1)	78 (48.8)	63 (95.5)	39 (97.5)
Unknown	27 (10.2)	7 (6.0)	2 (4.9)	13 (8.1)	3 (4.5)	1 (2.5)
ECOG PS						
0	165 (62.0)	76 (64.4)	28 (66.7)	95 (59.0)	36 (54.5)	22 (55.0)
1	44 (16.5)	15 (12.7)	8 (19.0)	36 (22.4)	19 (28.8)	8 (20.0)
≥ 2	10 (3.8)	2 (1.7)	0	1 (0.6)	1 (1.5)	1 (2.5)
Unknown	47 (17.7)	25 (21.2)	6 (14.3)	29 (18.0)	10 (15.2)	9 (22.5)
Visceral metastasis	128 (48.1)	62 (52.5)	24 (57.1)	90 (55.9)	41 (62.1)	29 (72.5)
Liver metastasis	51 (19.2)	17 (14.4)	4 (9.5)	44 (27.3)	18 (27.3)	11 (27.5)
Bone metastasis only	77 (28.9)	21 (17.8)	7 (16.7)	33 (20.5)	13 (19.7)	4 (10.0)
Disease-free interval^b^						
< 24 months	26 (9.8)	9 (7.6)	4 (9.5)	14 (8.7)	7 (10.6)	2 (5.0)
≥ 24 months	178 (66.9)	74 (62.7)	26 (61.9)	97 (60.2)	37 (56.1)	31 (77.5)
Treatment-free interval^c^						
De novo stage IV/Others^d^	60 (22.6)	37 (31.4)	14 (33.3)	47 (29.2)	21 (31.8)	7 (17.5)
< 12 months	138 (51.9)	44 (37.3)	13 (31.0)	68 (42.2)	20 (30.3)	17 (42.5)
≥ 12 months	49 (18.4)	26 (22.0)	12 (28.6)	31 (19.3)	14 (21.2)	9 (22.5)
Symptoms at palbociclib initiation^e^						
Yes	144 (54.1)	56 (47.5)	17 (40.5)	53 (32.9)	29 (43.9)	10 (25.0)
No	115 (43.2)	52 (44.1)	24 (57.1)	100 (62.1)	34 (51.5)	29 (72.5)
Unknown	7 (2.6)	10 (8.5)	1 (2.4)	8 (5.0)	3 (4.5)	1 (2.5)
Prior (neo-) adjuvant ET						
Yes	194 (72.9)	73 (61.9)	24 (57.1)	106 (65.8)	40 (60.6)	30 (75.0)
No	71 (26.7)	42 (35.6)	16 (38.1)	54 (33.5)	26 (39.4)	8 (20.0)
Unknown	1 (0.4)	3 (2.5)	2 (4.8)	1 (0.6)	0	2 (5.0)
Prior (neo-) adjuvant CT						
Yes	152 (57.1)	55 (46.6)	10 (23.8)	80 (49.7)	29 (43.9)	16 (40.0)
No	113 (42.5)	60 (50.8)	30 (71.4)	80 (49.7)	37 (56.1)	22 (55.0)
Unknown	1 (0.4)	3 (2.5)	2 (4.8)	1 (0.6)	0	2 (5.0)
Comorbidities						
Present	44 (16.5)	36 (30.5)	22 (52.4)	33 (20.5)	17 (25.8)	15 (37.5)
Absent	222 (83.5)	82 (69.5)	20 (47.6)	128 (79.5)	49 (74.2)	25 (62.5)
Type of comorbidity^f^						
Heart disease	8 (18.2)	7 (19.4)	8 (36.4)	6 (18.2)	3 (17.6)	6 (40.0)
Renal impairment^g^	2 (4.5)	5 (13.9)	2 (9.1)	3 (9.1)	1 (5.9)	1 (6.7)
Liver impairment^g^	4 (9.1)	1 (2.8)	1 (4.5)	4 (12.1)	2 (11.8)	1 (6.7)
Autoimmune disorders	9 (20.5)	6 (16.7)	2 (9.1)	3 (9.1)	2 (11.8)	2 (13.3)
Vascular disorders (including thromboembolism)	1 (2.3)	4 (11.1)	3 (13.6)	3 (9.1)	3 (17.6)	2 (13.3)
Other clinically significant comorbidities^h^	27 (61.4)	21 (58.3)	11 (50.0)	24 (72.7)	7 (41.2)	9 (60.0)

Data shown are n (%), unless otherwise specified

*AI* aromatase inhibitor;* CRF* case report form;* CT* chemotherapy;* ECOG PS* Eastern Cooperative Oncology Group Performance Status;* ET* endocrine therapy

^a^The denominator is the number of female patients

^b^Percentage was calculated based on patients with disease stage other than “stage IV”. The patients without the date of breast cancer surgery were excluded from this calculation

^c^Treatment-free interval was defined as the time from the end of adjuvant therapy to the diagnosis date of recurrence

^d^“Others” included patients who had surgery but did not undergo adjuvant therapy. The patients without the date of breast cancer surgery were excluded from this calculation

^e^Symptoms included bone pain, shortness of breath, coughing, headaches, dizziness, nausea, swelling around the neck and armpits, numbness in the limbs, abdominal bloating, and jaundice

^f^The denominator is the number of patients with a comorbidity

^g^As recorded in the CRF by physician judgment

^h^AI includes letrozole, anastrozole, and exemestane

### Real-world treatment pattern and dose modification of palbociclib by age group and treatment line

In the 1L setting, the palbociclib starting dose was 125 mg in 95.5%, 88.1%, and 64.3% of patients aged < 65 years, ≥ 65 to < 75 years, and ≥ 75 years, respectively (Table 2). There were no relevant differences across age groups in the 2L setting.

**Table 2:** Treatment patterns and dose modifications of palbociclib

	First-line (*N* = 426)			Second-line (*N* = 267)		
	< 65 years	≥ 65 to < 75 years	≥ 75 years	< 65 years	≥ 65 to < 75 years	≥ 75 years
	*n* = 266	*n* = 118	*n* = 42	*n* = 161	*n* = 66	*n* = 40
Initial palbociclib dose (mg/day)						
125	254 (95.5)	104 (88.1)	27 (64.3)	143 (88.8)	58 (87.9)	32 (80.0)
100	10 (3.8)	13 (11.0)	10 (23.8)	15 (9.3)	6 (9.1)	7 (17.5)
75	2 (0.8)	1 (0.8)	5 (11.9)	2 (1.2)	2 (3.0)	1 (2.5)
Other	0	0	0	1 (0.6)	0	0
Status of palbociclib administration						
Ongoing	66 (24.8)	21 (17.8)	6 (14.3)	16 (9.9)	8 (12.1)	8 (20.0)
Discontinued	200 (75.2)	97 (82.2)	36 (85.7)	145 (90.1)	58 (87.9)	32 (80.0)
Reason for discontinuation of palbociclib						
Adverse events	30 (11.3)	23 (19.5)	17 (40.5)	25 (15.5)	11 (16.7)	5 (12.5)
PD	155 (58.3)	63 (53.4)	13 (31.0)	113 (70.2)	44 (66.7)	25 (62.5)
Other	19 (7.1)	14 (11.9)	6 (14.3)	10 (6.2)	5 (7.6)	2 (5.0)
Reason for discontinuation of palbociclib treatment due to AEs						
Neutropenia	17 (6.4)	10 (8.5)	5 (11.9)	12 (7.5)	6 (9.1)	0
Febrile neutropenia	1 (0.4)	1 (0.8)	0	0	0	0
Leukopenia	3 (1.1)	2 (1.7)	1 (2.4)	3 (1.9)	0	0
Thrombocytopenia	0	3 (2.5)	1 (2.4)	3 (1.9)	1 (1.5)	0
Anemia	0	1 (0.8)	1 (2.4)	2 (1.2)	0	0
Non-hematologic toxicity	13 (4.9)	12 (10.2)	10 (23.8)	11 (6.8)	5 (7.6)	4 (10.0)
Other	2 (0.8)	2 (1.7)	2 (4.8)	1 (0.6)	1 (1.5)	1 (2.5)
Palbociclib dose reduction						
Yes	200 (75.2)	89 (75.4)	28 (66.7)	106 (65.8)	54 (81.8)	29 (72.5)
No	66 (24.8)	29 (24.6)	14 (33.3)	55 (34.2)	12 (18.2)	11 (27.5)
Timing of the first dose reduction of palbociclib						
≤ 3 months	153 (57.5)	71 (60.2)	25 (59.5)	78 (48.4)	47 (71.2)	27 (67.5)
> 3 to ≤ 6 months	22 (8.3)	11 (9.3)	1 (2.4)	14 (8.7)	3 (4.5)	0
> 6 to ≤ 9 months	7 (2.6)	3 (2.5)	0	5 (3.1)	1 (1.5)	0
> 9 months	18 (6.8)	4 (3.4)	2 (4.8)	9 (5.6)	3 (4.5)	2 (5.0)
Final dose after dose reduction for those receiving palbociclib (mg/day)						
100	68 (25.6)	32 (27.1)	6 (14.3)	45 (28.0)	19 (28.8)	7 (17.5)
75	122 (45.9)	56 (47.5)	17 (40.5)	57 (35.4)	33 (50.0)	21 (52.5)
Other	10 (3.8)	1 (0.8)	5 (11.9)	4 (2.5)	2 (3.0)	1 (2.5)
Type of ET						
Fulvestrant	153 (57.5)	64 (54.2)	23 (54.8)	124 (77.0)	52 (78.8)	30 (75.0)
AI^a^	109 (41.0)	53 (44.9)	18 (42.9)	37 (23.0)	13 (19.7)	8 (20.0)

Data shown are n (%), unless otherwise specified

*AE* adverse event;* ET* endocrine therapy;* PD* progressive disease

^a^AI includes letrozole, anastrozole, and exemestane

The rate of dose reductions was similar between age groups in the 1L setting (< 65 years: 75.2%; ≥ 65 to < 75 years: 75.4%; ≥ 75 years: 66.7%). In the 2L setting, patients aged ≥ 65 to < 75 years were most likely to experience dose reductions (81.8%) followed by those aged ≥ 75 years (72.5%) and < 65 years (65.8%).

The overall treatment discontinuation rate was slightly higher in patients aged ≥ 65 to < 75 years (82.2%) and ≥ 75 years (85.7%) compared with those aged < 65 years (75.2%) in the 1L setting. In the 1L setting, the most common reason for treatment discontinuation excluding PD was AEs (< 65 years: 11.3%; ≥ 65 to < 75 years: 19.5%; ≥ 75 years: 40.5%), followed by other reasons (< 65 years: 7.1%; ≥ 65 to < 75 years: 11.9%; ≥ 75 years: 14.3%). Overall, patients aged ≥ 75 years in the 1L setting were more likely to discontinue treatment due to AEs compared with other age groups, most commonly due to non-hematologic toxicity. Non-hematologic AEs leading to treatment discontinuation in patients aged ≥ 75 years in the 1L setting were rash (*n* = 2), decreased appetite (*n* = 2), interstitial lung disease (*n* = 1), pulmonary toxicity (*n* = 2), malaise (*n* = 1), depression (*n* = 1), hypoaesthesia (*n* = 1), palmar-plantar erythrodysaesthesia syndrome (*n* = 1), and pruritus (*n* = 1) (Table S1). Notably, in patients aged ≥ 75 years, treatment discontinuation was most common from 9 months after initiation. Discontinuation rates within the first 6 months were consistent across all age groups (Table S2).

### RwPFS, RwCFS and OS by age group and treatment line

**Fig. 1:**
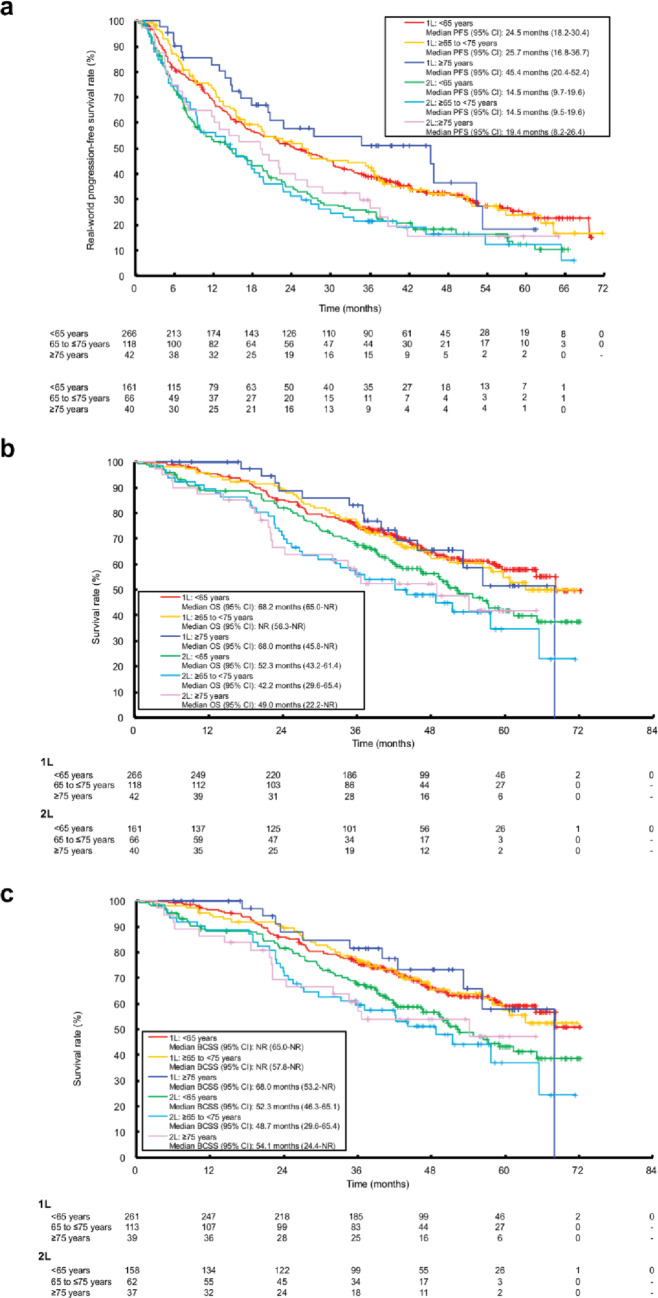
Real-world **a** PFS, **b** OS (all cause), and **c** breast cancer-specific survival with palbociclib plus ET as 1L and 2L treatment by treatment line and age group.* ET* endocrine therapy;* OS* overall survival;* PFS* progression-free survival;* 1L* first line;* 2L* second line

The mean (interquartile range [IQR]) follow-up time in the 1L treatment group from palbociclib initiation was 49.0 months (39.5–59.6) for patients aged < 65 years, 51.1 months (39.0-63.6) for those aged ≥ 65 to < 75 years, and 43.4 months (28.8–52.6) for those aged ≥ 75 years. In the 2L treatment group, corresponding median follow-up times were 48.4 months (38.8–62.1), 46.7 months (40.0–55.6) and 47.9 months (36.6–56.4), respectively. RwPFS, and OS, including overall survival and breast cancer-specific survival, are presented for each age group by treatment line in Fig. 1a–c. In the 1L treatment group, median rwPFS (95% CI) was 24.5 months (18.2–30.4), 25.7 months (16.8–36.7), and 45.4 months (20.4–52.4), in the < 65, ≥ 65 to < 75, and ≥ 75 years groups, respectively (Fig. 1a). In the 2L treatment group, corresponding median rwPFS (95% CI) was 14.5. months (9.7–19.6), 14.5 months (9.5–19.6), and 19.4 months (8.2–26.4), respectively (Fig. 1a).

**Fig. 2:**
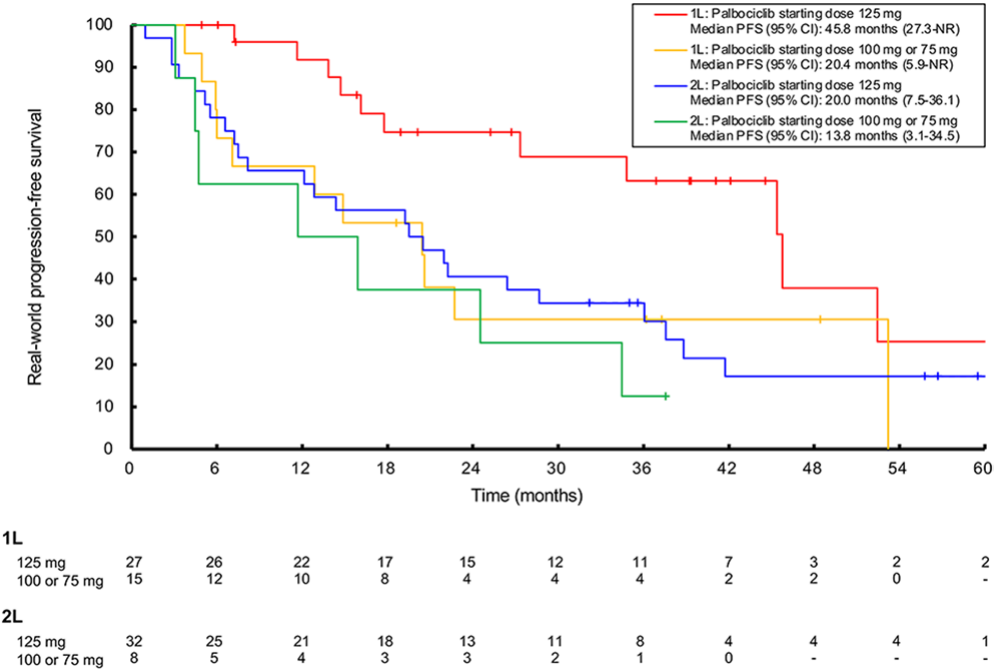
rwPFS in patients aged ≥ 75 years initiating palbociclib treatment at a starting dose of 125 mg versus 100 mg or 75 mg by treatment line and dose group

Patients aged ≥ 75 years who initiated palbociclib treatment in the 1L setting at 125 mg/day tended to show longer median rwPFS (45.8 months, 95%CI: 27.3-NR) compared with those who initiated palbociclib treatment at 100 mg/day or 75 mg/day (20.4 months, 95%CI: 5.9-NR; Fig. 2).

Median OS was similar across age groups in the 1L treatment setting, but there was a tendency towards longer OS in patients aged < 65 years in the 2L treatment setting (Fig. 1b). In the 1L treatment group, median OS (95% CI) was 68.2 months (65.0-NR) in patients aged < 65 years, NR (56.3-NR) in patients aged ≥ 65 to < 75 years, and 68.0 months (45.8-NR) in patients aged ≥ 75 years (Fig. 1b). In the 2L treatment group, median OS (95% CI) was 52.3 months (43.2–61.4) in patients aged < 65 years, 42.2 months (29.6–65.4) in patients aged ≥ 65 to < 75 years, and 49.0 (22.2-NR) in patients aged ≥ 75 years (Fig. 1b).

Exploratory analysis showed that median breast-cancer specific survival (95% CI) was similar among patients aged ≥ 75 years (68.1 months [53.2-NR]) and those aged < 65 years (NR [65.0-NR]) and ≥ 65 to < 75 years (NR [57.8-NR]) in the 1L setting, and slightly longer in patients aged ≥ 75 years (54.1 months [24.4-NR]) versus those aged < 65 years (52.3 months [46.3–65.1]) and ≥ 65 to < 75 years (48.7 months [29.6–65.4]) in the 2L setting (Fig. 1c). A total of 154 (36.2%) and 135 (50.6%) deaths occurred in 1L and 2L settings, respectively, with similar rates across age groups (1L, < 65 years: 35.3%; ≥ 65 to < 75 years: 39.0%; ≥ 75 years: 33.3%; 2L, < 65 years: 48.4%; ≥ 65 to < 75 years: 56.1%; ≥ 75 years: 50.0%). In 1L, breast cancer was the cause of death in 89 (94.7%) deaths aged < 65, 41 (89.1%) aged ≥ 65 to < 75, and 11 (78.6%) aged ≥ 75 years; other causes accounted for 1 (1.1%), 3 (6.5%), and 1 (7.1%) deaths, respectively. In 2L, breast cancer caused 75 (96.2%), 33 (89.2%), and 17 (85.0%) deaths in the same age groups; other causes accounted for 2 (2.6%), 4 (10.8%), and 1 (5.0%) deaths, respectively.

**Fig. 3:**
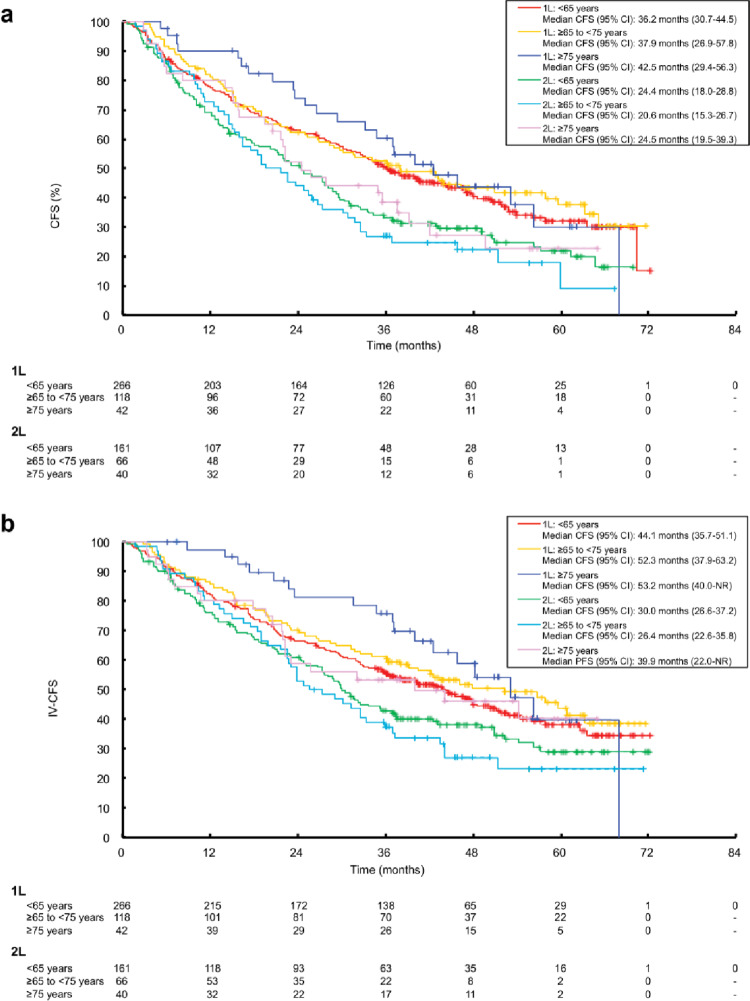
Analysis of **a** rwCFS, and **b** IV-rwCFS excluding oral fluoropyrimidine anticancer drugs, with palbociclib plus ET as 1L and 2L treatment by treatment line and age group.* ET* endocrine therapy;* IV* intravenous;* rwCFS* real-world chemotherapy-free survival;* 1L* first line;* 2L* second line

RwCFS was similar across all age groups in the 1L and 2L setting (Fig. 3a). Median rwCFS (95% CI) was 36.2 months (30.7–44.5) in patients aged < 65 years, 37.9 months (26.9–57.8) in patients aged ≥ 65 to < 75 years, and 42.5 months (29.4–56.3) in patients aged ≥ 75 years in the 1L setting. In the 2L setting, corresponding median rwCFS was 24.4 months (18.0-28.8), 20.6 months (15.3–26.7), and 24.5 months (19.5–39.3), respectively.

In both the 1L and 2L treatment setting, there was a tendency towards longer IV-rwCFS in patients aged ≥ 75 years compared with younger age groups (Fig. 3b). In the 1L treatment group, median IV-rwCFS (95% CI) was 44.1 months (35.7–51.1) in patients aged < 65 years, 52.3 months (37.9–63.2) in patients aged ≥ 65 to < 75 years, and 53.2 months (40.0-NR) in patients aged ≥ 75 years (Fig. 3b). In the 2L treatment group, median IV-rwCFS (95% CI) was 30.0 months (26.6–37.1) in patients aged < 65 years, 26.4 months (22.6–35.8) in patients aged ≥ 65 to < 75 years, and 39.9 months (22.0-NR) in patients aged ≥ 75 years (Fig. 3b).

In patients receiving subsequent therapy, ET-based therapy was more common in patients aged ≥ 75 years compared with younger age groups in both the 1L (80.0%) and 2L (66.7%) setting (Table 3). In patients aged ≥ 75 years, the most common ET regimen was ET monotherapy in both the 1L (40.0%) and 2L (26.7%) settings. In contrast, CT was more common in patients aged < 65 years (1L: 36.0%; 2L: 37.3%) compared with patients aged ≥ 75 years (1L: 20.0%; 2L: 33.3%) in both the 1L and 2L setting.

**Table 3:** Type of subsequent therapy^a^

	First-line (*N* = 426)			Second-line (*N* = 267)		
	< 65 years	≥ 65 to < 75 years	≥ 75 years	< 65 years	≥ 65 to < 75 years	≥ 75 years
	*n* = 266	*n* = 118	*n* = 42	*n* = 161	*n* = 66	*n* = 40
Patients without subsequent therapy	78 (29.3)	33 (28.0)	14 (33.3)	30 (18.6)	12 (18.2)	10 (25.0)
Continued treatment with palbociclib	65 (83.3)	21 (63.6)	5 (35.7)	14 (46.7)	8 (66.7)	8 (80.0)

Patients with subsequent therapy (denominator for subsequent treatments)	*n* = 186 (69.9%)	*n* = 84 (71.2%)	*n* = 25 (59.5%)	*n* = 126 (78.3%)	*n* = 53 (80.3%)	*n* = 30 (75.0%)
Type of first post-treatment regimen						
ET without CT	109 (58.6)	53 (63.1)	20 (80.0)	76 (60.3)	33 (62.3)	20 (66.7)
ET monotherapy	29 (15.6)	14 (16.7)	10 (40.0)	15 (11.9)	10 (18.9)	8 (26.7)
ET + CDK4/6 inhibitors	42 (22.6)	22 (26.2)	8 (32.0)	25 (19.8)	5 (9.4)	5 (16.7)
Palbociclib	1 (0.5)	1 (1.2)	0	2 (1.6)	2 (3.8)	0
Abemaciclib	41 (22.0)	21 (25.0)	8 (32.0)	23 (18.3)	3 (5.7)	5 (16.7)
ET + everolimus	37 (19.9)	16 (19.0)	2 (8.0)	36 (28.6)	18 (34.0)	7 (23.3)
ET + other	1 (0.5)	1 (1.2)	0 (0)	0 (0)	0 (0)	0 (0)
CT	67 (36.0)	29 (34.5)	5 (20.0)	47 (37.3)	20 (37.7)	10 (33.3)
CT + bevacizumab	28 (15.1)	7 (8.3)	0 (0)	11 (8.7)	3 (5.7)	0 (0)
CT (excluding oral fluoropyrimidine)	12 (6.5)	10 (11.9)	1 (4.0)	11 (8.7)	5 (9.4)	0 (0)
CT (oral fluoropyrimidine)	27 (14.5)	12 (14.3)	4 (16.0)	25 (19.8)	12 (22.6)	10 (33.3)
Other	10 (5.4)	2 (2.4)	0 (0)	3 (2.4)	0 (0)	0 (0)

Data shown are n (%), unless otherwise specified

*CT* chemotherapy;* ET* endocrine therapy;* NA* not applicable

^a^Denosumab, zoledronic acid, endoxan, goserelin, and leuprorelin were excluded from the tabulation

In patients receiving IV-CT as their subsequent therapy, exploratory analysis showed that median OS following IV-CT (95% CI) was 18.0 months (15.2–23.2) in patients aged < 65 years, 20.7 (15.3–23.5) in patients aged ≥ 65 to < 75 years, and NR (13.2-NR) in patients aged ≥ 75 years in the 1L treatment group (Fig. S2). In the 2L treatment group, median OS following IV-CT (95% CI) was 22.0 months (18.9–27.6) in patients aged < 65 years, 19.4 (14.3-NR) in patients aged ≥ 65 to < 75 years, and 8.9 (2.9–17.4) in patients aged ≥ 75 years (Fig. S2).

### Age-stratified analyses of patients initiating palbociclib at 125 mg/day

Baseline disease characteristics and outcomes (such as rwPFS, OS and CFS) of patients initiating palbociclib 125 mg/day analyzed by age group are shown in the Supplemental Tables and Figures. Patient backgrounds, rwPFS, OS, and CFS were largely similar between the overall population and patients who initiated palbociclib treatment at 125 mg/day (Table S3–S4, Fig. S3–S4).

## Discussion

Results of this subgroup analysis using real-world data from the retrospective, multicenter, observational P-BRIDGE study (*N* = 693) in Japan suggest that palbociclib plus ET is effective in patients with HR+/HER2- ABC, irrespective of age. RwPFS in the 1L setting was consistent with the range reported in RCTs [4, 5] and other real-world studies [12, 13]. Median OS was similar across age groups in the 1L treatment setting. Taken together, these data support the use of palbociclib + ET as a standard of care treatment in younger and elderly Japanese patients with HR+/HER2- ABC.

The non-significant tendency towards longer median rwPFS in patients aged ≥ 75 years in 1L compared with younger age groups may be influenced by the small sample size for the ≥ 75 year age subgroup and differences in patient background characteristics, including the higher proportion of patients with a TFI of ≥ 12 months and de novo stage IV disease in this subgroup. Therefore, this finding in the elderly age group warrants further investigation in future analyses. Nevertheless, the results of the present subgroup analyses are generally consistent with the results of several prior studies, which have demonstrated the effectiveness of palbociclib in older patients with HR+/HER2- ABC [11–13, 15–17]. In a pooled analysis of the PALOMA clinical studies exploring the efficacy and safety of palbociclib ± ET in patients with HR+/HER2- ABC aged ≥ 65 years, the median PFS was longer in patients aged 65 to 74 years (HR [95% CI]: 0.66 [0.45–0.97]; *p* = 0.016) and ≥ 75 years (HR [95% CI]: 0.31 [0.16–0.61]; *p* < 0.001) receiving palbociclib plus letrozole versus letrozole alone in PALOMA-1 and -2 [11]. In a subanalysis of patients aged ≥ 75 years in the retrospective, observational P-REALITY X study of the Flatiron Health Analytic Database [12], the median rwPFS in the palbociclib + aromatase inhibitor (AI) group was longer than in the AI group (HR [95% CI]: 0.72 [0.59–0.89]; *p* = 0.0021) after stabilized inverse probability treatment weighting. Corresponding median OS rates were similar to rwPFS (HR [95% CI: 0.66 [0.51–0.84]; *p* = 0.0007).

Despite patients aged ≥ 75 years having a higher rate of comorbidities compared with those aged ≥ 65 to < 75 years and < 65 years (45.1%, 18.0%, and 28.8%, respectively) in our sub-analysis, no consistent differences in OS were observed between older and younger age groups. Additionally, breast cancer-specific survival also tended to be largely similar between treatment groups and age groups. These results suggest that palbociclib is an attractive option when considering treatment strategies for elderly Japanese patients with diverse backgrounds.

Compared with previous reports, the rate of treatment discontinuation due to AEs overall and specifically due to non-hematologic toxicity was higher in elderly patients receiving 1L palbociclib in P-BRIDGE (40.5% and 23.8%, respectively) [13, 17–19]. In our study, loss of appetite, rash, and pneumonia occurred in 4.8% of cases. Aside from these events, the incidence of other events was less than 3%, and there were no non-hematologic toxicities occurring in > 3% of patients, which were more common in the elderly. The pooled analysis of the PALOMA clinical studies showed that discontinuation rates were similar across all age groups [11]. In Cohort A (ET-sensitive and 1L treatment for ABC) of the French prospective PalomAGE study in patients aged ≥ 70 years, the discontinuation rate due to AEs was 7.7% [19]. Hematologic toxicities are considered the main adverse drug reactions leading to treatment discontinuation during palbociclib combination therapy. However, discontinuations due to non-hematologic toxicities occurred in 23.8% of patients aged ≥ 75 years in the 1L setting in our study, which was higher than in other age groups (< 65 years: 4.9%; ≥ 65 to < 75 years: 10.2%). This finding shows that the management of non-hematologic toxicities is an important consideration in the elderly cohort.

In our subgroup analysis of P-BRIDGE, patients aged ≥ 75 years were more likely to be commenced on a lower initial palbociclib dose compared with those aged < 75 years. A high rate of comorbidities in the ≥ 75-year age group may partially explain why dose reductions were initiated more frequently at the discretion of the attending physician in this patient group. While patient backgrounds differed, those aged ≥ 75 years who commenced therapy at the 125 mg dose tended to have longer rwPFS than patients who started on a reduced dose (i.e. 100 mg or 75 mg) despite performance status (PS) being comparable to that of other age groups. In the overall population, factors such as TFI, presence of liver metastases, and PS were associated with differences in PFS. Notably, within the ≥ 75-year subgroup, the proportion of patients with a TFI ≥ 12 months and de novo disease was higher in the group that initiated treatment at 125 mg (Table S5). To account for this imbalance, we conducted an additional analysis stratified by TFI. Even after this analysis, patients who started at 125 mg still showed a trend toward better outcomes, supporting the appropriateness of this starting dose in selected elderly patients. These findings, which are similar to those of the PalomAGE study, highlight the necessity of performing a comprehensive geriatric assessment and tailoring treatment approaches based on each patient’s condition rather than relying solely on chronological age, and those who are determined as being able to commence therapy on 125 mg should be encouraged to do so where possible [20]. Results of the PalomAGE study further showed that factors such as social support should be implemented where possible as they may influence treatment continuation and patient outcomes [20]. Taken together, these findings suggest that careful examination of the patient’s condition, appropriate AE management considering patients’ age and comorbidities, and support from allied health professionals are important for maximizing treatment outcomes with palbociclib in the elderly.

In the open-label, phase 3 SELECT-BC trial conducted across 154 hospitals in Japan, S-1 was non-inferior to taxane with respect to OS as a 1L treatment for metastatic breast cancer and demonstrated significantly better quality of life (QOL) than taxane drugs [21]. These findings have contributed to a preference for oral chemotherapy over IV options in patients aged ≥ 75 years, particularly as a means to preserve QOL and delay the need for IV treatment. When selecting potential treatment options, it is important to consider those that maintain QOL and delay the need for IV CT in addition to prolonging PFS and OS. This is particularly true for elderly patients, who often experience reduced physical and physiological resilience. In this context, the time to initiation of intravenous chemotherapy, as measured by CFS, is a relevant clinical consideration. RwCFS in patients receiving IV-CT tended to be longer in those aged ≥ 75 years compared with younger age groups in both the 1L and 2L treatment setting (Fig. 3b). Specifically, median IV-rwCFS was 53.2 months in patients aged ≥ 75 years versus 44.1 months in those aged < 65 years in the 1L group, and 39.9 months versus 30.0 months in the 2L setting (Fig. 3b). Although interpretation of OS following IV-CT in patients aged ≥ 75 years was limited due to the small sample size, OS following IV-CT was comparable among patients aged < 65 years and those aged ≥ 65 to < 75 years (Fig. S2). These findings underscore the importance of treatments that preserve QOL and delay the initiation of IV chemotherapy, particularly in older patients. As some patients may benefit from earlier treatment with IV-CT, it is important to ensure that treatment opportunities are not missed through flexible decision-making based on individual patients. This exploratory OS analysis should be interpreted with caution due to significant confounding factors. The cases included in this analysis were selected based on physicians’ treatment decisions, which introduces significant limitations.

Since this study did not evaluate economic burden, the societal impact of medical costs fell outside the scope of this analysis. Importantly, our findings demonstrate that palbociclib plus ET is effective regardless of age, reinforcing its potential as a viable treatment option for elderly patients. In alignment with the ESMO/SIOG Cancer in the Elderly Working Group position paper [22], treatment decisions should be guided by a comprehensive geriatric assessment and tailored to the individual characteristics of each patient. In Japan, however, rising pharmaceutical expenditure for the elderly has emerged as an urgent societal concern, driven largely by the burgeoning ageing population. The adoption of innovative drugs such as CDK4/6 inhibitors in the elderly may be influenced not only by the cost of these treatments but the need for caregiver support [20]. Consequently, treatment selection for elderly patients must involve a comprehensive evaluation that considers not only treatment effectiveness but also the patient’s condition, economic circumstances [23], and care environment.

This subanalysis from the P-BRIDGE study has several potential limitations. The lack of a control arm (e.g. ET monotherapy) and relatively short duration of follow-up caused limitations in the assessment of time-to-event outcomes. P-BRIDGE was a retrospective study using data retrieved from electronic medical records, which may include missing or erroneous data. Disease progression was not based on standard criteria (e.g., Response Evaluation Criteria in Solid Tumors), but instead on the individual treating physician’s clinical assessment and interpretation of radiographic or pathologic results. This study only collected AE data in relation to discontinuations; therefore, it was not possible to evaluate the overall safety of palbociclib in elderly patients from this study in Japan. Furthermore, this study was conducted in Japanese patients and was limited to study sites with experience prescribing palbociclib to ≥ 10 patients in both the 1L and 2L settings; therefore, the results may not be generalizable to patient populations in other countries or clinics treating fewer than 10 patients. Finally, the sample size of patients aged ≥ 75 years was particularly small in both the 1L (*n* = 42) and 2L (*n* = 40) setting, which may have influenced the results.

In conclusion, this subgroup analysis from the P-BRIDGE study underlines the clinical benefits of palbociclib plus ET in patients with HR+/HER2- ABC in Japan. No differences in effectiveness were observed between elderly patients and younger patients with respect to survival outcomes. Elderly patients were more likely to start on a reduced dose and discontinue treatment due to non-hematologic toxicities. However, for patients who are able to initiate therapy at the full 125 mg dose, it is important to perform a comprehensive assessment and avoid reducing the dose based solely on advanced age, given the associated PFS benefits. Moreover, our study highlights the need for careful monitoring and appropriate management in this cohort.

## Supplementary Information

Below is the link to the electronic supplementary material.

Supplementary Material 1
