# Supplementary material for: Real-world outcomes of palbociclib plus endocrine therapy in elderly patients with HR+/HER2- advanced breast cancer in Japan: a subgroup analysis of the P-BRIDGE study by age group
Source: Breast Cancer. 2026 Feb 11;33(2):333–45. doi: 10.1007/s12282-025-01812-5 (PMC12960296; doi:10.1007/s12282-025-01812-5)

# Supplementary Appendix

# Table S1. Summary of non-hematologic adverse events leading to discontinuation of palbociclib by treatment line and age

|  | **First-line (N=426)** | | | **Second-line (N=267)** | | |
| --- | --- | --- | --- | --- | --- | --- |
|  | **<65 years** | **≥65 to <75 years** | **≥75 years** | **<65 years** | **≥65 to <75 years** | **≥75 years** |
|  | **N=266** | **N=118** | **N=42** | **N=161** | **N=66** | **N=40** |
| Non-hematologic toxicity |  |  |  |  |  |  |
| Pneumonia | 1 (0.4) | 1 (0.8) | 0 | 0 | 0 | 0 |
| Decreased appetite | 0 | 0 | 2 (4.8) | 0 | 0 | 0 |
| Depression | 0 | 0 | 1 (2.4) | 0 | 0 | 0 |
| Headache | 1 (0.4) | 0 | 0 | 0 | 0 | 0 |
| Hypoaesthesia | 0 | 0 | 1 (2.4) | 0 | 0 | 0 |
| Tinnitus | 1 (0.4) | 0 | 0 | 0 | 0 | 0 |
| Arrythmia | 0 | 0 | 0 | 1 (0.6) | 0 | 0 |
| Pericardial effusion | 0 | 0 | 0 | 1 (0.6) | 0 | 0 |
| Cough | 0 | 1 (0.8) | 0 | 0 | 0 | 0 |
| Dyspnoea | 0 | 0 | 0 | 1 (0.6) | 0 | 0 |
| Interstitial lung disease | 3 (1.1) | 3 (2.5) | 1 (2.4) | 1 (0.6) | 1 (1.5) | 1 (2.5) |
| Pleural effusion | 0 | 0 | 0 | 1 (0.6) | 0 | 0 |
| Pulmonary toxicity | 0 | 0 | 2 (4.8) | 0 | 0 | 0 |
| Abdominal pain upper | 0 | 0 | 0 | 1 (0.6) | 0 | 0 |
| Gastroesophageal reflux disease | 0 | 1 (0.8) | 0 | 0 | 0 | 0 |
| Nausea | 0 | 1 (0.8) | 0 | 1 (0.6) | 0 | 0 |
| Obstruction gastric | 0 | 1 (0.8) | 0 | 0 | 0 | 0 |
| Stomatitis | 1 (0.4) | 0 | 0 | 1 (0.6) | 1 (1.5) | 0 |
| Vomiting | 0 | 0 | 0 | 1 (0.6) | 0 | 0 |
| Hepatic function abnormal | 2 (0.8) | 0 | 0 | 1 (0.6) | 2 (3.0) | 0 |
| Liver disorder | 1 (0.4) | 0 | 0 | 0 | 0 | 0 |
| Drug-induced liver injury | 0 | 0 | 0 | 0 | 1 (1.5) | 0 |
| Palmar-plantar erythrodysaesthesia syndrome | 0 | 0 | 1 (2.4) | 0 | 0 | 0 |
| Pruritus | 0 | 1 (0.8) | 1 (2.4) | 0 | 0 | 0 |
| Rash | 2 (0.8) | 1 (0.8) | 2 (4.8) | 0 | 0 | 1 (2.5) |
| Urticaria | 0 | 0 | 0 | 0 | 0 | 1 (2.5) |
| Renal disorder | 0 | 0 | 0 | 1 (0.6) | 0 | 0 |
| Acute kidney injury | 0 | 1 (0.8) | 0 | 0 | 0 | 0 |
| Malaise | 1 (0.4) | 2 (1.7) | 1 (2.4) | 2 (1.2) | 1 (1.5) | 1 (2.5) |
| Pyrexia | 2 (0.8) | 0 | 0 | 1 (0.6) | 0 | 0 |
| Inflammation | 0 | 0 | 0 | 1 (0.6) | 0 | 0 |
| C-reactive protein increased | 0 | 1 (0.8) | 0 | 0 | 0 | 0 |

Data shown are n (%), unless otherwise specified.

# Table S2. Time to discontinuation of palbociclib due to adverse events

|  | **First-line (N=70)** | | | **Second-line (N=41)** | | |
| --- | --- | --- | --- | --- | --- | --- |
|  | **<65  years** | **≥65 to <75 years** | **≥75  years** | **<65  years** | **≥65 to <75 years** | **≥75  years** |
|  | **n=30** | **n=23** | **n=17** | **n=25** | **n=11** | **n=5** |
| Time from first administration of palbociclib to discontinuation of palbociclib due to AEs, n (%) | | | | | | |
| ≤3 months | 11 (36.7) | 10 (43.5) | 4 (23.5) | 9 (36.0) | 7 (63.6) | 2 (40.0) |
| >3 to ≤6 months | 6 (20.0) | 3 (13.0) | 6 (35.3) | 2 (8.0) | 2 (18.2) | 1 (20.0) |
| >6 to ≤9 months | 5 (16.7) | 4 (17.4) | 0 | 4 (16.0) | 0 | 1 (20.0) |
| >9 months | 8 (26.7) | 6 (26.1) | 7 (41.2) | 10 (40.0) | 2 (18.2) | 1 (20.0) |

AE, adverse event.

# Table S3. Patient demographics and clinical characteristics of patients receiving palbociclib 125 mg

|  | **First-line (N=385)** | | | **Second-line (N=233)** | | |
| --- | --- | --- | --- | --- | --- | --- |
|  | **<65 years** | **≥65 to <75 years** | **≥75 years** | **<65 years** | **≥65 to <75 years** | **≥75 years** |
|  | **n=254** | **n=104** | **n=27** | **n=143** | **n=58** | **n=32** |
| Sex |  |  |  |  |  |  |
| Male | 1 (0.4) | 1 (1.0) | 0 | 1 (0.7) | 0 | 0 |
| Female | 253 (99.6) | 103 (99.0) | 27 (100.0) | 142 (99.3) | 58 (100.0) | 32 (100.0) |
| Menopausal status^a^ |  |  |  |  |  |  |
| Pre/perimenopausal | 84 (33.2) | 0 | 0 | 61 (43.0) | 0 | 0 |
| Postmenopausal | 143 (56.5) | 96 (93.2) | 26 (96.3) | 69 (48.6) | 55 (94.8) | 31 (96.9) |
| Unknown | 26 (10.3) | 7 (6.8) | 1 (3.7) | 12 (8.5) | 3 (5.2) | 1 (3.1) |
| ECOG PS |  |  |  |  |  |  |
| 0 | 158 (62.2) | 65 (62.5) | 18 (66.7) | 88 (61.5) | 30 (51.7) | 18 (56.3) |
| 1 | 42 (16.5) | 13 (12.5) | 5 (18.5) | 31 (21.7) | 17 (29.3) | 5 (15.6) |
| ≥2 | 10 (3.9) | 2 (1.9) | 0 | 1 (0.7) | 1 (1.7) | 0 |
| Unknown | 44 (17.3) | 24 (23.1) | 4 (14.8) | 23 (16.1) | 10 (17.2) | 9 (28.1) |
| Visceral metastasis | 121 (47.6) | 55 (52.9) | 16 (59.3) | 79 (55.2) | 37 (63.8) | 24 (75.0) |
| Liver metastasis | 47 (18.5) | 15 (14.4) | 2 (7.4) | 38 (26.6) | 16 (27.6) | 11 (34.4) |
| Bone metastasis only | 74 (29.1) | 18 (17.3) | 4 (14.8) | 32 (22.4) | 12 (20.7) | 2 (6.3) |
| Disease-free interval^b^ |  |  |  |  |  |  |
| <24 months | 26 (10.2) | 7 (6.7) | 1 (3.7) | 11 (7.7) | 6 (10.3) | 1 (3.1) |
| ≥24 months | 168 (66.1) | 70 (67.3) | 15 (55.6) | 86 (60.1) | 32 (55.2) | 28 (87.5) |
| Treatment-free interval^c^ |  |  |  |  |  |  |
| De novo stage IV/Others | 58 (22.8) | 30 (28.8) | 12 (44.4) | 42 (29.4) | 19 (32.8) | 3 (9.4) |
| <12 months | 132 (52.0) | 38 (36.5) | 4 (14.8) | 62 (43.4) | 17 (29.3) | 14 (43.8) |
| ≥12 months | 46 (18.1) | 26 (25.0) | 8 (29.6) | 25 (17.5) | 11 (19.0) | 8 (25.0) |
| Symptoms at palbociclib initiation^d^ |  |  |  |  |  |  |
| Yes | 140 (55.1) | 51 (49.0) | 12 (44.4) | 49 (34.3) | 26 (44.8) | 8 (25.0) |
| No | 107 (42.1) | 44 (42.3) | 15 (55.6) | 87 (60.8) | 30 (51.7) | 23 (71.9) |
| Unknown | 7 (2.8) | 9 (8.7) | 0 | 7 (4.9) | 2 (3.4) | 1 (3.1) |
| Prior (neo-) adjuvant ET |  |  |  |  |  |  |
| Yes | 187 (73.6) | 67 (64.4) | 12 (44.4) | 95 (66.4) | 34 (58.6) | 26 (81.3) |
| No | 67 (26.4) | 34 (32.7) | 13 (48.1) | 47 (32.9) | 24 (41.4) | 4 (12.5) |
| Unknown | 0 | 3 (2.9) | 2 (7.4) | 1 (0.7) | 0 | 2 (6.3) |
| Prior (neo-) adjuvant CT |  |  |  |  |  |  |
| Yes | 143 (56.3) | 51 (49.0) | 5 (18.5) | 72 (50.3) | 25 (43.1) | 14 (43.8) |
| No | 111 (43.7) | 50 (48.1) | 20 (74.1) | 70 (49.0) | 33 (56.9) | 16 (50.0) |
| Unknown | 0 | 3 (2.9) | 2 (7.4) | 1 (0.7) | 0 | 2 (6.3) |
| Comorbidities |  |  |  |  |  |  |
| Present | 44 (17.3) | 34 (32.7) | 15 (55.6) | 27 (18.9) | 13 (22.4) | 12 (37.5) |
| Absent | 210 (82.7) | 70 (67.3) | 12 (44.4) | 116 (81.1) | 45 (77.6) | 20 (62.5) |
| Type of comorbidity^e^ |  |  |  |  |  |  |
| Heart disease | 8 (18.2) | 7 (20.6) | 5 (33.3) | 3 (11.1) | 2 (15.4) | 5 (41.7) |
| Renal dysfunction | 2 (4.5) | 5 (14.7) | 2 (13.3) | 2 (7.4) | 1 (7.7) | 1 (8.3) |
| Liver dysfunction | 4 (9.1) | 0 | 1 (6.7) | 4 (14.8) | 2 (15.4) | 0 |
| Autoimmune disorders | 9 (20.5) | 6 (17.6) | 1 (6.7) | 2 (7.4) | 1 (7.7) | 1 (8.3) |
| Vascular disorders (including thromboembolism) | 1 (2.3) | 4 (11.8) | 2 (13.3) | 0 | 2 (15.4) | 2 (16.7) |
| Other clinically significant comorbidities^f^ | 27 (61.4) | 20 (58.8) | 8 (53.3) | 20 (74.1) | 6 (46.2) | 7 (58.3) |

Data shown are n (%), unless otherwise specified.

CT, chemotherapy; ECOG PS, Eastern Cooperative Oncology Group Performance Status; ET, endocrine therapy.

^a^The denominator is the number of female patients

^b^Percentage was calculated based on patients with disease stage other than “stage IV”. The patients without the date of breast cancer surgery were excluded from this calculation

^c^“Others” included patients who had surgery but did not undergo adjuvant therapy. The patients without the date of breast cancer surgery were excluded from this calculation

^d^Symptoms included bone pain, shortness of breath, coughing, headaches, dizziness, nausea, swelling around the neck and armpits, numbness in the limbs, abdominal bloating, and jaundice

^e^The denominator is the number of patients with a comorbidity.

^f^AI includes letrozole, anastrozole, and exemestane.

# Table S4. Treatment patterns and dose modifications in patients receiving palbociclib 125 mg plus ET as 1L and 2L treatment by treatment line and age group

|  | **First-line (N=385)** | | | **Second-line (N=233)** | | |
| --- | --- | --- | --- | --- | --- | --- |
|  | **<65 years** | **≥65 to <75 years** | **≥75 years** | **<65 years** | **≥65- to <75 years** | **≥75 years** |
|  | **n=254** | **n=104** | **n=27** | **n=143** | **n=58** | **n=32** |
| Initial palbociclib dose (mg/day) |  |  |  |  |  |  |
| 125 | 254 (100.0) | 104 (100.0) | 27 (100.0) | 143 (100.0) | 58 (100.0) | 32 (100.0) |
| 100 | 0 | 0 | 0 | 0 | 0 | 0 |
| 75 | 0 | 0 | 0 | 0 | 0 | 0 |
| Other | 0 | 0 | 0 | 0 | 0 | 0 |
| Status of palbociclib administration |  |  |  |  |  |  |
| Ongoing | 61 (24.0) | 17 (16.3) | 2 (7.4) | 15 (10.5) | 8 (13.8) | 7 (21.9) |
| Discontinued | 193 (76.0) | 87 (83.7) | 25 (92.6) | 128 (89.5) | 50 (86.2) | 25 (78.1) |
| Reason for discontinuation of palbociclib |  |  |  |  |  |  |
| Adverse events | 28 (11.0) | 21 (20.2) | 14 (51.9) | 23 (16.1) | 10 (17.2) | 5 (15.6) |
| PD | 150 (59.1) | 55 (52.9) | 6 (22.2) | 99 (69.2) | 38 (65.5) | 19 (59.4) |
| Other | 19 (7.5) | 13 (12.5) | 5 (18.5) | 9 (6.3) | 4 (6.9) | 1 (3.1) |
| Reason for discontinuation of palbociclib treatment due to AEs |  |  |  |  |  |  |
| Neutropenia | 17 (6.7) | 8 (7.7) | 3 (11.1) | 11 (7.7) | 6 (10.3) | 0 |
| Febrile neutropenia | 1 (0.4) | 1 (1.0) | 0 | 0 | 0 | 0 |
| Leukopenia | 3 (1.2) | 1 (1.0) | 1 (3.7) | 3 (2.1) | 0 | 0 |
| Thrombocytopenia | 0 | 2 (1.9) | 1 (3.7) | 3 (2.1) | 1 (1.7) | 0 |
| Anemia | 0 | 1 (1.0) | 1 (3.7) | 2 (1.4) | 0 | 0 |
| Non-hemat toxicity | 11 (4.3) | 12 (11.5) | 9 (33.3) | 11 (6.8) | 5 (7.6) | 4 (10.0) |
| Other | 2 (0.8) | 2 (1.9) | 2 (7.4) | 1 (0.7) | 1 (1.7) | 1 (3.1) |
| Palbociclib dose reduction |  |  |  |  |  |  |
| Yes | 195 (76.8) | 81 (77.9) | 20 (74.1) | 99 (69.2) | 49 (84.5) | 25 (78.1) |
| No | 59 (23.2) | 23 (22.1) | 7 (25.9) | 44 (30.8) | 9 (15.5) | 7 (21.9) |
| Timing of the first dose reduction of palbociclib |  |  |  |  |  |  |
| ≤3 months | 149 (58.7) | 64 (61.5) | 18 (66.7) | 74 (51.7) | 42 (72.4) | 23 (71.9) |
| >3 to ≤6 months | 21 (8.3) | 10 (9.6) | 0 | 11 (7.7) | 3 (5.2) | 0 |
| >6 to ≤9 months | 7 (2.8) | 3 (2.9) | 0 | 5 (3.5) | 1 (1.7) | 0 |
| >9 months | 18 (7.1) | 4 (3.8) | 2 (7.4) | 9 (6.3) | 3 (5.2) | 2 (6.3) |
| Final dose after dose reduction for those receiving palbociclib (mg/day) |  |  |  |  |  |  |
| 100 | 68 (26.8) | 32 (30.8) | 6 (22.2) | 44 (30.8) | 19 (32.8) | 7 (21.9) |
| 75 | 118 (46.5) | 48 (46.2) | 11 (40.7) | 51 (35.7) | 28 (48.3) | 17 (53.1) |
| Other | 9 (3.5) | 1 (1.0) | 3 (11.1) | 4 (2.8) | 2 (3.4) | 1 (3.1) |
| Type of ET |  |  |  |  |  |  |
| Fulvestrant | 146 (57.5) | 54 (51.9) | 14 (51.9) | 112 (78.3) | 46 (79.3) | 25 (78.1) |
| AI^a^ | 104 (40.9) | 50 (48.1) | 13 (48.1) | 31 (21.7) | 11 (19.0) | 6 (18.8) |

Data shown are n (%), unless otherwise specified.

AE, adverse event; ET, endocrine therapy; PD, progressive disease

^a^AI includes letrozole, anastrozole, and exemestane.

# Table S5. Patient demographics and clinical characteristics of patients aged ≥75 years in the 1L setting initiating palbociclib at a starting dose of 125 mg versus 100 mg or 75 mg

|  | **First-line treatment,**  **≥75 years (N=42)** | |
| --- | --- | --- |
|  | **Palbociclib starting dose** | |
|  | **125 mg** | **100 mg or 75 mg** |
|  | **n=27** | **n=15** |
| Sex |  |  |
| Male | 0 | 1 (6.7) |
| Female | 27 (100.0) | 14 (93.3) |
| Menopausal status^a^ |  |  |
| Pre/perimenopausal | 0 | 0 |
| Postmenopausal | 26 (96.3) | 13 (92.9) |
| Unknown | 1 (3.7) | 1 (7.1) |
| ECOG PS |  |  |
| 0 | 18 (66.7) | 10 (66.7) |
| 1 | 5 (18.5) | 3 (20.0) |
| ≥2 | 0 | 0 |
| Unknown | 4 (14.8) | 2 (13.3) |
| Visceral metastasis | 16 (59.3) | 8 (53.3) |
| Liver metastasis | 2 (7.4) | 2 (13.3) |
| Bone metastasis only | 4 (14.8) | 3 (20.0) |
| Disease-free interval^b^ |  |  |
| <24 months | 1 (3.7) | 3 (20.0) |
| ≥24 months | 15 (55.6) | 11 (73.3) |
| Treatment-free interval^c^ |  |  |
| De novo stage IV/Others^d^ | 12 (44.4) | 2 (13.3) |
| <12 months | 4 (14.8) | 9 (60.0) |
| ≥12 months | 8 (29.6) | 4 (26.7) |
| Symptoms at palbociclib initiation^e^ |  |  |
| Yes | 12 (44.4) | 5 (33.3) |
| No | 15 (55.6) | 9 (60.0) |
| Unknown | 0 | 1 (6.7) |
| Prior (neo-) adjuvant ET |  |  |
| Yes | 12 (44.1) | 12 (80.0) |
| No | 13 (48.1) | 3 (20.0) |
| Unknown | 2 (7.4) | 0 |
| Prior (neo-) adjuvant CT |  |  |
| Yes | 5 (18.5) | 5 (33.3) |
| No | 20 (74.1) | 10 (66.7) |
| Unknown | 2 (7.4) | 0 |
| Comorbidities |  |  |
| Present | 15 (55.6) | 7 (46.7) |
| Absent | 12 (44.4) | 8 (53.3) |
| Type of comorbidity^f^ |  |  |
| Heart disease | 5 (33.3) | 3 (42.9) |
| Renal dysfunction | 2 (13.3) | 0 |
| Liver dysfunction | 1 (6.7) | 0 |
| Autoimmune disorders | 1 (6.7) | 1 (14.3) |
| Vascular disorders (including thromboembolism) | 2 (13.3) | 1 (14.3) |
| Other clinically significant comorbidities^g^ | 8 (53.3) | 3 (42.9) |

Data shown are n (%), unless otherwise specified.

CT, chemotherapy; ECOG PS, Eastern Cooperative Oncology Group Performance Status; ET, endocrine therapy.

^a^The denominator is the number of female patients

^b^Percentage was calculated based on patients with disease stage other than “stage IV”. The patients without the date of breast cancer surgery were excluded from this calculation

^c^Treatment-free interval was defined as the time from the end of adjuvant therapy to the diagnosis date of recurrence.

^d^“Others” included patients who had surgery but did not undergo adjuvant therapy. The patients without the date of breast cancer surgery were excluded from this calculation.

^e^Symptoms included bone pain, shortness of breath, coughing, headaches, dizziness, nausea, swelling around the neck and armpits, numbness in the limbs, abdominal bloating, and jaundice

^f^The denominator is the number of patients with a comorbidity.

^g^AI includes letrozole, anastrozole, and exemestane.

# Figure S1. Patient flow diagram
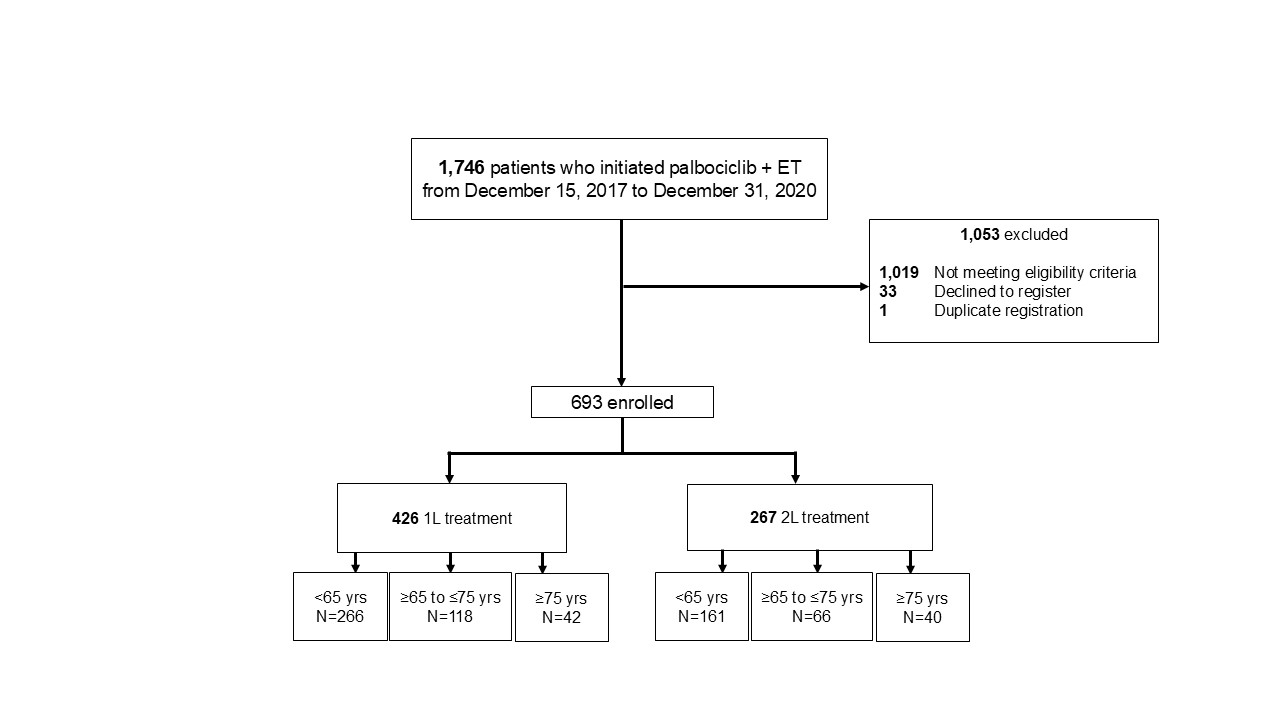


ET, endocrine therapy; 1L, first line; 2L, second line

# Figure S2. Exploratory analysis of OS following IV-CT in patients treated with palbociclib plus ET as 1L and 2L treatment by treatment line and age group


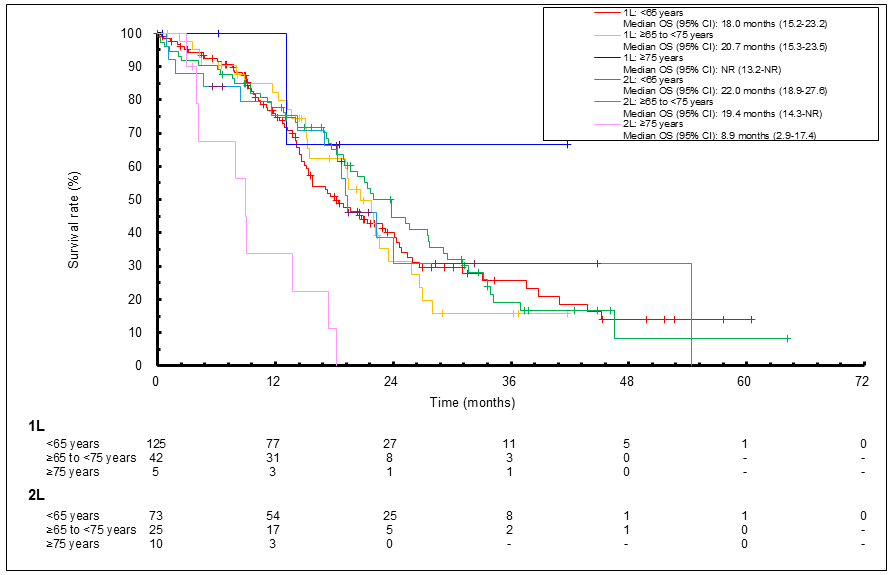


ET, endocrine therapy; IV-CT, intravenous chemotherapy; OS, overall survival; 1L, first line; 2L, second line

# Figure S3. Real-world (a) PFS and (b) OS (all cause) in patients receiving palbociclib 125 mg plus ET as 1L and 2L treatment by treatment line and age group

ET, endocrine therapy; OS, overall survival; PFS, progression-free survival; 1L, first line; 2L, second line

(a)
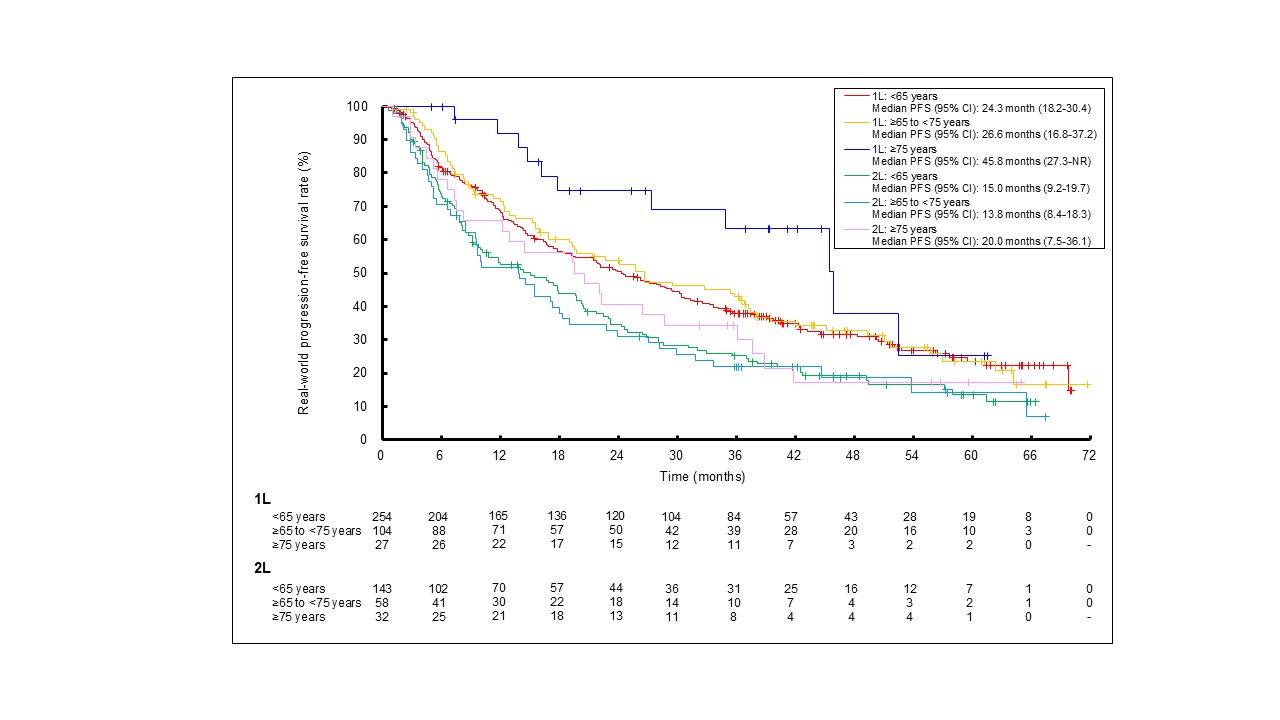


(b)

**
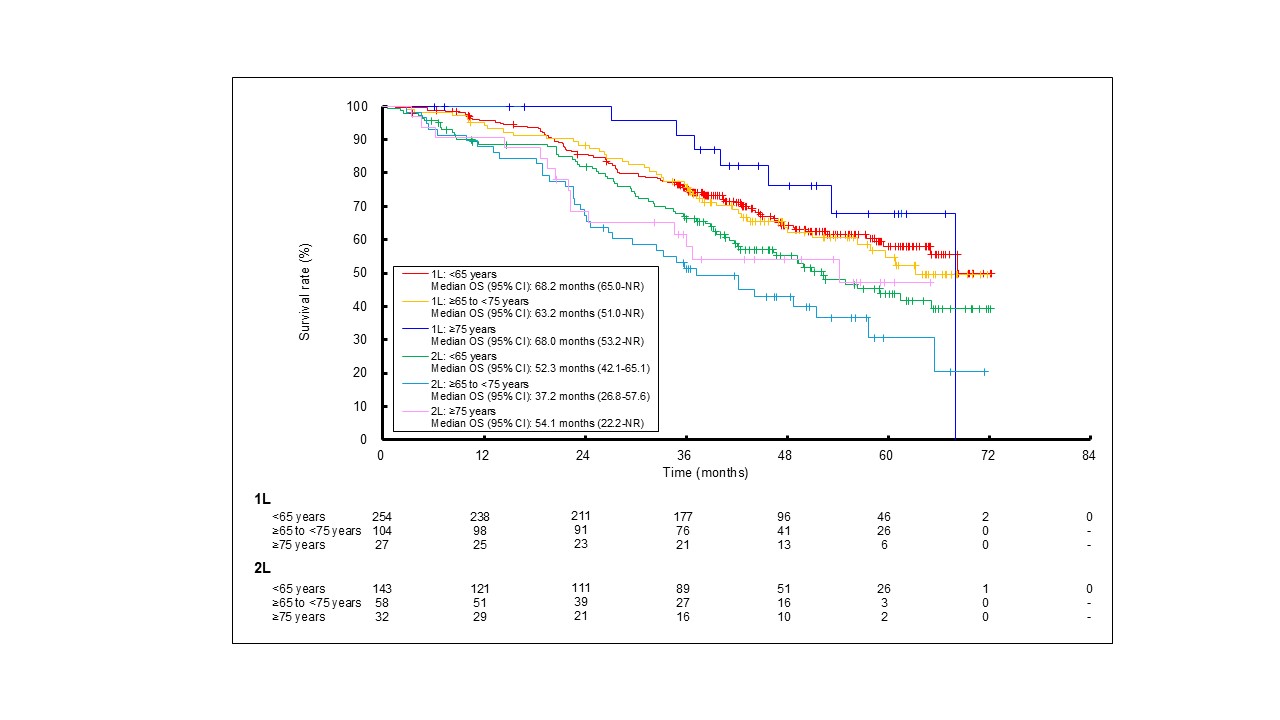
**

# Figure S4. Analysis of (a) CFS, and (b) CFS excluding oral fluoropyrimidine anticancer drugs, in patients receiving palbociclib 125 mg plus ET as 1L and 2L treatment according to treatment line and age

(a)


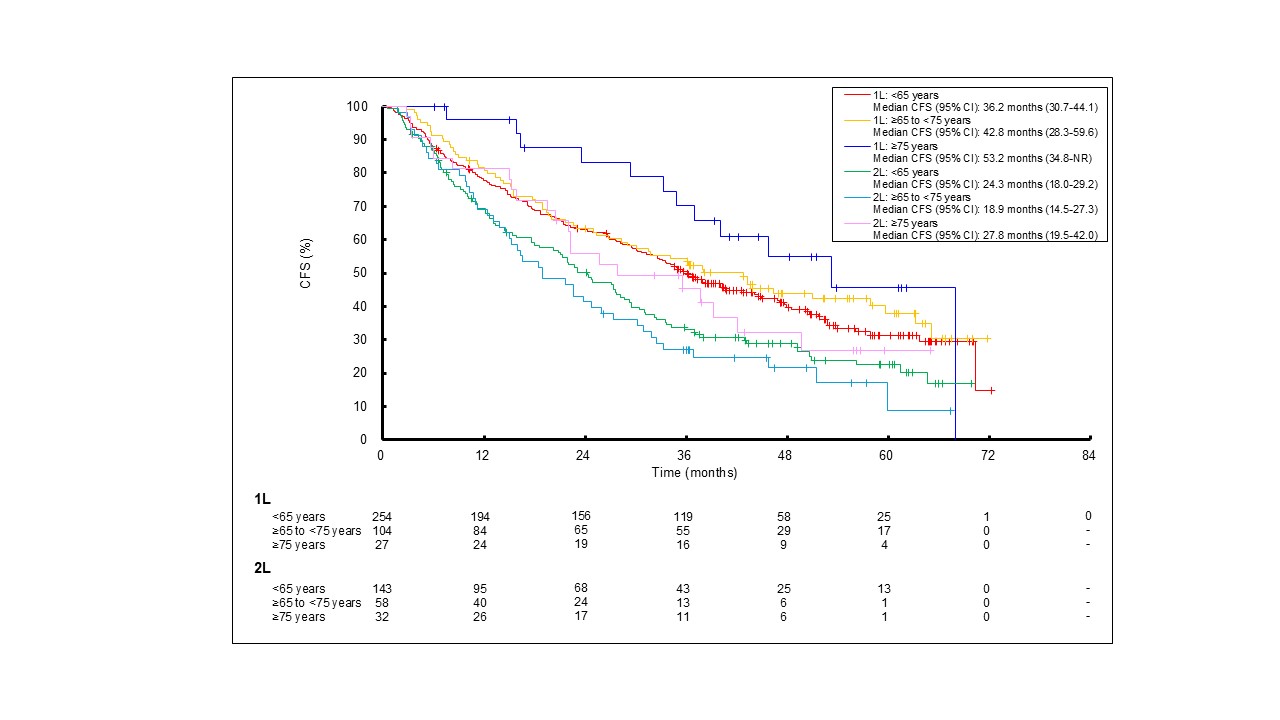


CFS, chemotherapy-free survival; ET, endocrine therapy; 1L, first line; 2L, second line

(b)


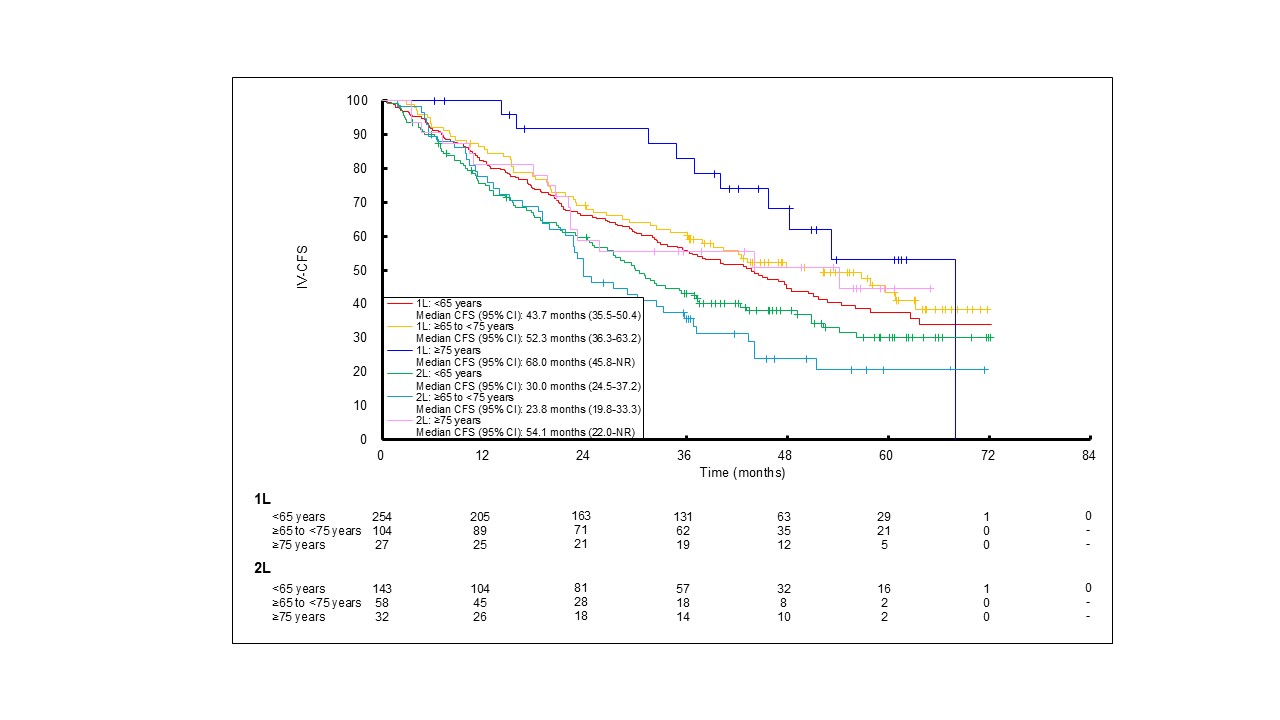

Supplement: Supplementary file 1 — Supplementary Material 1 [file 12282_2025_1812_MOESM1_ESM.docx]
